# Low Doses of Arsenic in a Mouse Model of Human Exposure and in Neuronal Culture Lead to S-Nitrosylation of Synaptic Proteins and Apoptosis via Nitric Oxide

**DOI:** 10.3390/ijms21113948

**Published:** 2020-05-31

**Authors:** Haitham Amal, Guanyu Gong, Hongmei Yang, Brian A. Joughin, Xin Wang, Charles G. Knutson, Maryam Kartawy, Igor Khaliulin, John S. Wishnok, Steven R. Tannenbaum

**Affiliations:** 1 Institute for Drug Research, School of Pharmacy, Faculty of Medicine, The Hebrew University of Jerusalem, Jerusalem 91120, Israel; maryam.kartawy@mail.huji.ac.il (M.K.); igorkh@savion.huji.ac.il (I.K.); 2 Department of Biological Engineering, Massachusetts Institute of Technology, Cambridge, MA 02139, USA; gongguanyu2010@hotmail.com (G.G.); yanghm@mit.edu (H.Y.); bjoughin@mit.edu (B.A.J.); xinw@mit.edu (X.W.); cgfknutson@hotmail.com (C.G.K.); wishnok@mit.edu (J.S.W.); 3 Koch Institute for Integrative Cancer Research, Massachusetts Institute of Technology, Cambridge, MA 02139, USA; 4 Department of Chemistry, Massachusetts Institute of Technology, Cambridge, MA 02139, USA

**Keywords:** Arsenic, nitric oxide, S-nitrosylation, brain disorders, brain cortex, mouse, nitrosative stress, apoptosis, synaptic processes, acetyl-CoA

## Abstract

Background: Accumulating public health and epidemiological literature support the hypothesis that arsenic in drinking water or food affects the brain adversely. Methods: Experiments on the consequences of nitric oxide (NO) formation in neuronal cell culture and mouse brain were conducted to probe the mechanistic pathways of nitrosative damage following arsenic exposure. Results: After exposure of mouse embryonic neuronal cells to low doses of sodium arsenite (SA), we found that Ca^2+^ was released leading to the formation of large amounts of NO and apoptosis. Inhibition of NO synthase prevented neuronal apoptosis. Further, SA led to concerted S-nitrosylation of proteins significantly associated with synaptic vesicle recycling and acetyl-CoA homeostasis. Our findings show that low-dose chronic exposure (0.1–1 ppm) to SA in the drinking water of mice led to S-nitrosylation of proteomic cysteines. Subsequent removal of arsenic from the drinking water reversed the biochemical alterations. Conclusions: This work develops a mechanistic understanding of the role of NO in arsenic-mediated toxicity in the brain, incorporating Ca^2+^ release and S-nitrosylation as important modifiers of neuronal protein function.

## 1. Introduction

Living in areas contaminated with arsenic can affect the cognitive development of children. For example, Rosado et al. investigated the cognitive parameters (memory, attention, problem solving, and vocabulary processes) in 6–8-year-old children living near a metallurgic smelter in Mexico with high exposure to arsenic and lead. The scientists found that children with an arsenic level in urine exceeding 50 μg/L had significantly weaker cognitive abilities than those with a urine arsenic concentration of below 50 μg/L [1]. Another large-scale observational study has shown that autistic spectrum disorder prevailed in census tracts located in the areas polluted with arsenic, lead, and mercury [2]. An association was also found between lower IQ in children and exposure to arsenic in drinking water [3]. A community-based participatory research of people subjected to groundwater arsenic exposure (current and long-term) revealed a significant correlation to poorer scores in cognition in adults [4]. Arsenic is found globally in water supplies and affects more than 140 million people worldwide. Domestically, some water supplies in Nevada, New Hampshire, and the Midwest exceed the drinking water threshold of the World Health Organization and the United States Environmental Protection Agency (EPA) (10 ppb) [5]. Exposure to high arsenic via drinking water was found also in China (4 ppm), Taiwan (3 ppm), India (0.8 ppm), and other countries [5]. Arsenic has recently come under scrutiny for its concentration in baby food, particularly in rice and rice products [6]. Previous studies have shown that exposure to arsenic in drinking water could induce neurological and cognitive dysfunctions [7,8,9,10]. Thus, experiments on rats exposed to arsenic at the concentration of 68 mg/L in drinking water have demonstrated spatial memory damage due to morphological and biochemical abnormalities in hippocampus [9]. In another study, post-natal exposure of rats to low-level arsenic (45 μg/L) resulted in impaired learning and social skills, increased anxiety-like behaviors, and abnormal frontal cortex neurogenesis [10]. Arsenic-exposed (at 50 ppb) mice had significantly lower levels of corticosterone receptors and considerably affected learning and memory behavior [8]. Similar results have been shown in the epidemiological studies on children and adults consuming water with low concentrations of arsenic. For example, Tyler and Allan have reported in their review about the abnormal alterations in neurogenesis, as well as in Ras-MAPK/ERK, cholinergic, and monoaminergic signaling, which negatively affect the cognitive functions [7]. However, the underlying mechanisms of these changes are unknown and the current understanding of the impact of arsenic on the central nervous system remains poor, particularly in terms of its molecular mechanism.

Nitric oxide (NO) is a signaling molecule in the central and peripheral nervous system and is one of the most important messenger molecules [11,12]. NO has a critical role in synaptic transmission in the brain [12] and plays a major role in signaling through the activation of GMP cyclase [12], effects on ion transporters [12], and S-nitrosylation (SNO) of peptides and proteins [13,14]. Numerous examples of neuropathologies exist that involve nitrosative stress (for review see [15,16]), including Alzheimer’s [17], Parkinson’s [18] and Huntington’s diseases [19]. In addition, we have recently demonstrated the involvement of NO, SNO, and nitration in a mouse model of autism based on the *SHANK3* human mutation [20].

Here, we tested whether low-dose chronic exposure of arsenic, similar to levels found in water in different regions in the world [5], induces NO-molecular changes in the brain cortical regions and whether transitioning arsenic-watered mice to arsenic-free water for 10 days would reverse the differential SNO. Finally, we conducted proteomic analysis to test SNO of proteins in the cortex of arsenic-watered mice and to examine which biological processes and signaling pathways are affected by the S-nitrosylated proteins.

## 2. Results

### 2.1. Arsenic-Induced Apoptosis Was Mediated by Ca^2+^ Release and NO

**Figure 1:**
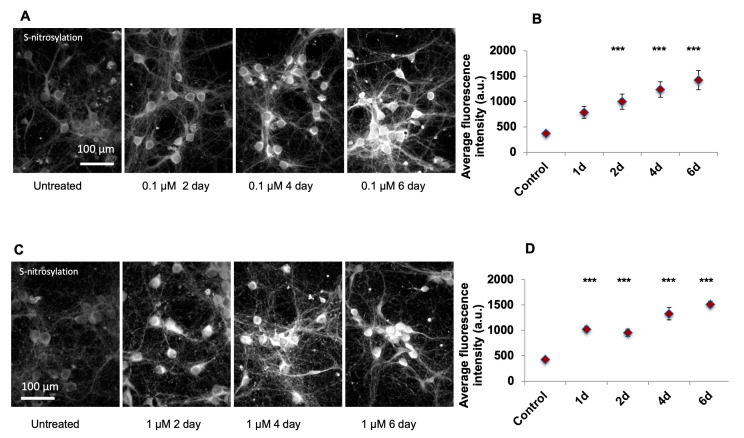
Primary cultures of mouse embryonic cortical neurons were used to test S-nitrosylation. (**A**) S-nitrosylation (SNO)-proteins were stained using SNOTRAP conjugated with a fluorophore (Cy5) following sodium arsenite (SA) 0.1 µM treatment. (**B**) The differences in SNO following treatment with 0.1 µM SA were measured for 1–6 days. Significant (***) upregulations were found from 1st day of exposure when compared to untreated. (**C**) SNO-proteins following 1 µM SA treatment were stained using SNOTRAP. (**D**) The differences in SNO following treatment with 1 µM SA were measured for 1–6 days, Significant (***) upregulations were found from the 1st day of exposure when compared to untreated cells. All quantification is presented as mean ± SEM. *p* < 0.0005 ***. One-way ANOVA followed by Tukey post hoc test of treatment (each day) vs. untreated group of cells was conducted. 1 µM NaAs = 0.13 ppm.

**Figure 2:**
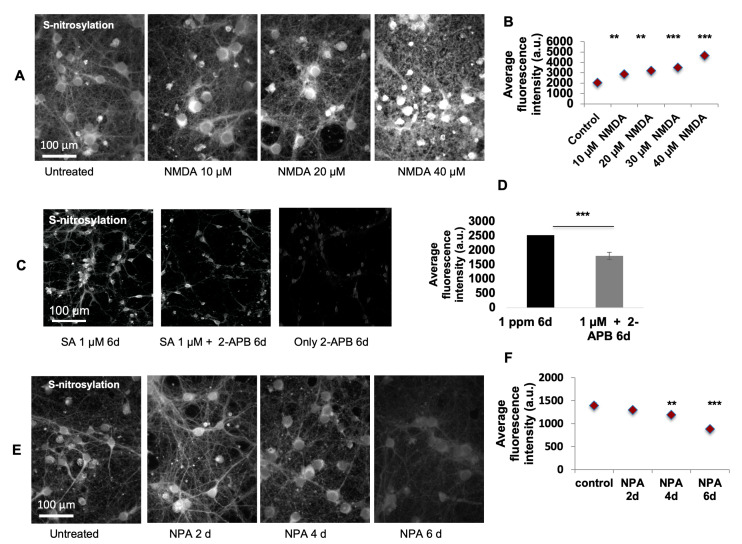
Pharmacological experiments with primary cultures of mouse embryonic cortical neurons were used to understand the molecular mechanism. (**A**) Cells were treated with NMDA for 24 h, leading to upregulation of SNOTRAP staining. (**B**) The differences in SNO following treatment with 10, 20, 30, or 40 μM of NMDA were measured. Significant (***) upregulations were found following all exposures. (**C**) A Ca^2+^ channel blocker, 2-Aminoethoxydiphenyl borate (2-APB) was added to the primary cultured neurons treated with 1 µM SA and without treatment. Representative staining of SNO before and after 2-APB treatment is shown. (**D**) The level of SNO was measured. A significant reduction in SNO was found following 2-APB (100 µM) treatment (**). (**E**) Cells were treated with neuronal NOS inhibitor, N(ω)-propyl-l-arginine (NPA), for 6 days. Representative staining is shown. (**F**) The differences in SNO following treatment with NPA (2 μM) were measured for 6 days. Significant reduction of SNO staining was found on the 4th (**) and 6th (***) day following the treatment. All quantification is presented as mean ± SEM. *p* < 0.005 **, *p* < 0.0005 ***. One-way ANOVA followed by Tukey post hoc test of treatment (each dose/day) vs. untreated group of cells was conducted. 1 µM NaAs = 0.13 ppm.

**Figure 3:**
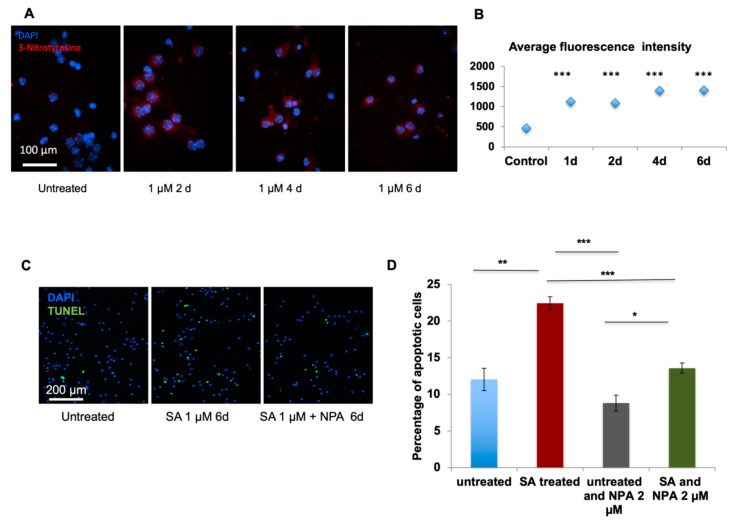
Primary cultures of mouse embryonic cortical neurons were used to test Ntyr and apoptosis. (**A**) Representative staining of 3-nitrotyrosine (red) and DAPI (blue) before and after SA 1 µM treatment, (**B**) The differences in 3-nitrotyrosine following treatment with SA 1 µM were measured for 6 days. A significant upregulation was found (***) following treatment for 1–6 days. (**C**) Cell apoptosis was measured before and after 1 µM SA and NPA treatments using the Terminal deoxynucleotidyl transferase (TdT) dUTP nick-end labeling (TUNEL) assay. (**D**) Percentage of apoptotic cells was calculated. A significant increase in apoptosis following 1 µM SA treatment (**) compared to untreated mice. A significant reduction was found following treatment with 1 µM SA and NPA (***) compared to SA -treated cells. All quantification is presented as mean ± SEM. *p* < 0.05 *, *p* < 0.005 **, *p* < 0.0005 ***. One-way ANOVA followed by Tukey post hoc test of treatment in each day vs. untreated was conducted for Ntyr quantification. Two-Way ANOVA was conducted for apoptosis quantification. 1 µM NaAs = 0.13 ppm.

In order to develop a mechanistic understanding of the effect of arsenic in the brain, we performed in vitro experiments on mouse embryonic cortical neurons in culture. Low levels of SA (0.1 µM) led to instantaneous Ca^2+^ release from the cell body, and Ca^2+^ saturated the synapse in ~5 s (Figure S1 and Movie S1). Next, we measured levels of S-nitrosylation [21] following SA exposure. Immunofluorescence analysis of a chemical probe for SNO-proteins (SNOTRAP)-labeled cells was done and one-way ANOVA demonstrates a significant increase of SNO in SA-treated cells in the 0.1 µM (F_4,45_ = 18.09) (Figure 1A,B) when compared to the untreated time point at the 2nd day (*p* < 0.0001), 4th day (*p* < 0.0001), and 6th day (*p* < 0.0001) of the treatment. One-way ANOVA reveals a significant increase of SNO in SA-treated cells in the 1 µM (F_4,90_ = 18.79) when comparing untreated time to the 1st day (*p* < 0.001), 2nd day (*p* < 0.001), 4th day (*p* < 0.001), and 6th day (*p* < 0.001) of treatment (Figure 1C,D) SNO-positive cells were neuronal in structure (Figure 2A) and SNO-proteins were distributed throughout the neuronal cell with much lower amounts in dendrocytes. To develop a molecular mechanistic understanding of SNO differences, we conducted several pharmacological experiments. The SNO signal was dramatically upregulated after NMDA treatment to activate glutamate channels (Figure 2A,B). ANOVA analysis reveals significant increase of untreated cells compared to treated with different doses of NMDA (F_5,325_ = 22.34); significant differences were found between untreated vs. 10 µM (*p* < 0.001), 20 µM (*p* < 0.001), 30 µM (*p* < 0.0001), and 40 µM (*p* < 0.0001) treatments. The Ca^2+^ channel blocker 2-Aminoethoxydiphenyl borate (2-APB) significantly (F_1,29_ = 19.36, *p* < 0.001) prevented the increase of SNO (Figure 2C,D). Treatment with the nNOS inhibitor N(ω)-propyl-l-arginine (NPA) significantly down-regulated the SNO signal (F_3,60_ = 22.73) when compared untreated cells to 4th day (*p* < 0.001) and 6th day (*p* < 0.0001) of treatment (Figure 2E,F). Next, we measured nitration and found a significant upregulation in 3-nitrotyrosine (Ntyr) following 1 µM SA treatment (Figure 3A,B), but not after 0.1 µM SA treatment (Figure S3A,B). One-way ANOVA reveals a significant upregulation of Ntyr in SA-treated cells in the 1 µM (F_4,55_ = 237.3) when comparing untreated cells to 1st day (*p* < 0.0001), 2nd day (*p* < 0.0001), 4th day (*p* < 0.0001), and 6th day (*p* < 0.0001) of treatment (Figure 3A,B). A significant increase in apoptosis is found only at the higher dose of 1 µM of SA (F_3,100_ = 45.92, *p* < 0.0001) (Figure 3C,D), but not following 0.1 µM SA exposure (Figure S3C,D). Finally, to test whether apoptosis is mediated via NO, as mentioned above, we treated the cells with NPA, and strikingly, we find a significant decrease (F_3,100_ = 45.92, *p* < 0.0001) in apoptosis, to approximately the baseline level, in cells not treated with SA (Figure S3C,D).

### 2.2. The Brains of Arsenic-Watered Mice Upregulated Levels of GSNO and 3-Nitrotyrosine

**Figure 4:**
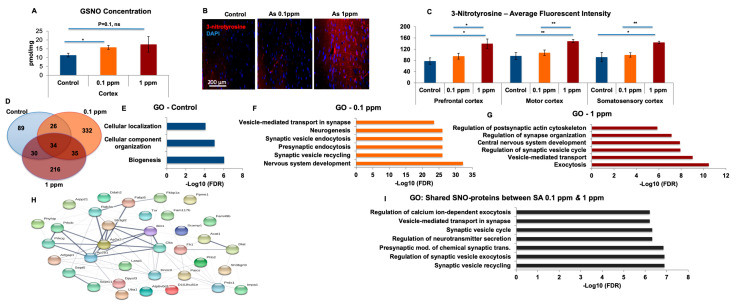
Changes in (nitric oxide) NO and SNO biochemistry in-vivo (**A**) S-Nitrosoglutathione (GSNO) concentrations were measured in the cortex of control mice and mice watered with 0.1 ppm or 1 ppm SA. The multiple reaction monitoring (MRM) mode of a triple quadrupole mass spectrometer (QqQ MS) was used to establish a sensitive and selective quantification method. Y-axis represents the concentration of GSNO in pmol/mg protein. There was a significant upregulation in the SA 0.1 ppm and 1 ppm mice compared to the control mice. Each group is *n* = 3. (**B**) Representative staining of 3-nitrotyrosine (red) and DAPI (blue) in control, 0.1 ppm SA, and 1 ppm SA. (**C**) The average fluorescent intensity of 3-nitrotyrosine of the three groups in different cortical regions: prefrontal cortex, motor cortex and somatosensory cortex. A significant upregulation in 1 ppm SA compared to 0.1 ppm and control was found in all regions. Each group was *n* = 8. All quantifications are presented as mean ± SEM. One tailed t-tests were conducted. *p* < 0.05 *, *p* < 0.005 **. (**D**) A Venn diagram shows the number of SNO-proteins in each group. Gene ontology (GO) analysis of the SNO-proteins following the treatment exclusively found in (**E**) control, (**F**) 0.1 ppm SA, and (**G**) 1 ppm SA. (**H**) Interactome analysis of the 35 proteins (**I**) GO analysis of the proteins overlapped exclusively between 0.1 ppm and 1 ppm SA groups following the treatment. Bars represent the –log10 of the Benjamini-Hochberg corrected false discovery rate (FDR). Abbreviations: mod.: modulation; trans.: transmission.

To validate our NO biochemical findings, and to identify specific S-nitrosylated proteins in vivo, we used a mouse model of human exposure to arsenic (see the Introduction). Mice were exposed to 0.1 and 1 ppm SA in drinking water for a month. Beforehand, we measured the concentration of SA in the cortices of treated and control mice to confirm SA uptake and found a significant upregulation in treated mice (Figure S4). First, we quantified S-nitrosoglutathione (GSNO) in the brains of these mice. Using chemical probe-based mass spectrometry (MS) for targeted GSNO quantification [22], we found a statistically significant upregulation of GSNO in the cortices of the SA-treated mice compared to the controls (Figure 4A). Next, we examined brain regions [23] with potential elevation of Ntyr levels. Ntyr in the prefrontal, motor, and somatosensory cortical regions were significantly upregulated in the 1 ppm SA treatment group (Figure 4B,C) relative to controls.

### 2.3. S-Nitrosylation of Proteins in the Cortex of Arsenic-Watered Mice Occured Preferentially on Proteins Involved in Synaptic Biology

We used SNOTRAP [21] in combination with MS to identify specific S-nitrosylated proteins in SA-treated mice. We found differences in the identities of the SNO-proteins between SA-treated mice and controls and between the 0.1 ppm and 1 ppm groups (Figure 4D). Identities of the SNO-proteins are shown in Table S1.

To gain a systems-level insight into SNO-proteins’ functionalities, we conducted a Gene Ontology (GO) analysis of the SNO-proteomes of each treatment group to identify biological processes and pathway modifications enriched relative to the background of the total proteome. Proteins associated mostly with basic cellular and metabolic processes were SNOed in the control group (Figure 4E, Tables S1 and S2). However, in the SA-treated groups, synaptic processes linked to signaling, including synaptic vesicle recycling, exocytosis, and endocytosis in pre- and post-synaptic regions were affected (Figure 4F,G, Table S2). A Venn diagram was constructed (Figure 4D) in order to identify critical proteins shared only between the 0.1 and 1 ppm doses of SA, and not controls. Only 35 proteins met these criteria (Table S3). They were further analyzed in STRING (Figure 4H) and KEGG for biological processes and pathway analysis, respectively. We found that these proteins are critical for regulating many synaptic processes and the cholinergic system (Figure 4I, Table S3). Interactome analysis (Figure 4H) of the SNO-proteins of SA treated mice shows a cluster of “acetyl-CoA metabolic process” proteins (*p* < 0.0005), which generates the precursor for acetylcholine. In this cluster, mitochondrial Acetyl-CoA-acetyltransferase was SNOed in mice watered with both 0.1 and 1 ppm SA on the catalytic subunit cysteine 410.

### 2.4. Arsenic-Watered Mice Showed Reversible NO-Molecular Alterations

**Figure 5:**
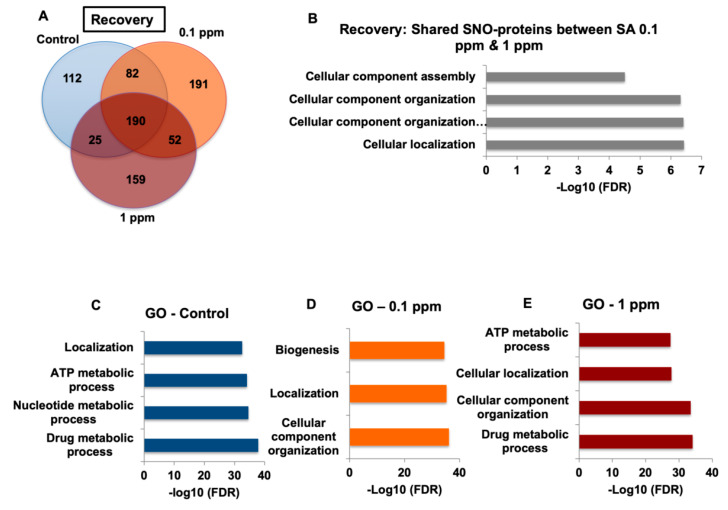
Recovery experiments to test NO and SNO reversibility in-vivo (**A**) A Venn diagram shows the number of SNO-proteins in each group following recovery. (**B**) Gene ontology (GO) analysis of the proteins exclusively overlapped between 0.1 ppm and 1 ppm SA following the recovery period. (C-D) GO analysis of the SNO-proteins following the recovery period found exclusively in (**C**) control, (**D**) 0.1 ppm SA, and (**E**) 1 ppm SA. Bars represent the –log10 of the Benjamini-Hochberg corrected false discovery rate (FDR).

Finally, to test for reversibility in the biochemistry results upon withdrawal of SA, we proceeded with a mouse model of human exposure to arsenic followed by 10 days on SA-free water. In these experiments, the same two groups of juvenile 3-week-old mice drinking water with two different low levels of SA (0.1 ppm and 1 ppm) and one control group with SA-free water for a month were employed. The SNOTRAP-based MS analysis was conducted following the recovery period to identify the SNO-proteome in the cortices of the three groups. We found that the number of shared SNO-proteins in the three groups after the recovery (see Figure 5A) was higher than following the treatment (Figure 4D), whilst the number of exclusive proteins in each group was lower after the recovery. We searched for the 35 proteins shared between the SA groups found following the treatment. None of the 35 SNO-proteins critical for modification of the key synaptic processes, or for maintenance of acetyl CoA homeostasis were found in the SA reversal groups (Figure 5B). Further, GO analysis of SNO proteins of each group after a recovery period showed none of the neuronal and synaptic processes that were found following the treatment (Figure 5C,E). The goal was to present qualitative analysis, and the results were expressed only as present or absent. As shown above, this analysis was followed by systems biology analysis to identify aggregates of observations that point to the same direction with respect to the families of proteins with commonalities of function that were identified. We further conducted a direct comparison of high dose treatment vs. recovery and low dose treatment vs. recovery. We excluded the overlapped proteins and analyzed the proteins exclusively identified in each of these groups. For example, exocytosis process was found in both low and high dose treatments and were not found in the recovery groups. The non-canonical Wnt signaling pathway, which is associated with Alzheimer’s disease [17], was found following treatment with 0.1 ppm but not following recovery. In conclusion, transitioning SA-watered mice to SA-free water for 10 days reverses the differential S-nitrosylation.

## 3. Discussion

**Figure 6:**
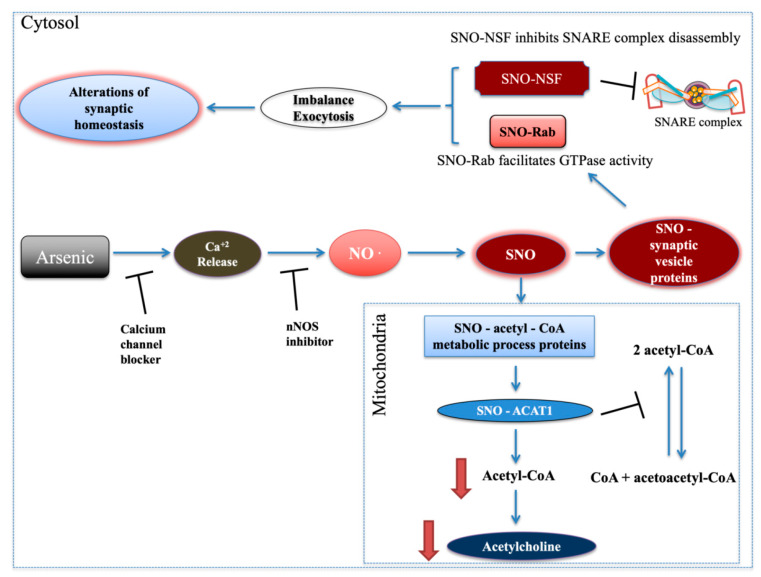
Schematic model summarizing the in vivo and in vitro findings. Low dose of arsenic led to increase of Ca^2+^ release and NO formation, which were inhibited by calcium channel blocker and nNOS inhibitor, respectively. Mostly, synaptic vesicle proteins were SNOed. SNO-NSF and SNO-Rab might lead to alterations in synaptic homeostasis. Proteins associated with acetylcholine system were SNOed and might lead to decrease of acetylcholine production, which is associated with different behavioral pathologies. Abbreviations: NO: nitric oxide; SNO: S-nitrosylation. ACAT1: Acetyl-CoA acetyltransferase, mitochondrial.

This work combines biochemical and cellular experiments both in vitro and in vivo, develops mechanistic understanding, and proves a role for NO in the arsenic-mediated neuronal apoptosis. The results suggest the involvement of calcium, NO, and apoptosis in the mechanism of cognitive behavior alteration in arsenic-treated mice. Brain arsenic in drinking water had a dose-dependent effect, demonstrating that the brain-blood barrier (BBB) does not effectively block the passage of arsenic to the CNS [24]. Previous studies have shown that NO can mediate neurotoxicity, leading to neurological disorders [25,26,27]. Since Ca^2+^/calmodulin is one of the main regulators of nNOS activity [23], we first tested whether Ca^2+^ is released from cellular stores following arsenic exposure [28], leading to NO generation and the resultant S-nitrosylation and nitration of proteins [23]. We hypothesize that SA functions by targeting IP3/Ry receptors, promoting calcium release, and the calcium efflux activates the nNOS and NO production. Our in-vitro findings are supported by recent pioneering publications that have implicated NO [27,29], calcium signaling [30], and apoptosis [31] as possible molecular mechanisms for behavioral pathologies. Note that since Ca^2+^, [30] NO, and Ntyr [32] are indicators of neuronal damage, we followed up by assessing the degree of apoptosis. Generally, NO has a critical role in synaptic transmission in the brain [12] and plays a major role in signaling through SNO of peptides and proteins [13,14]. As a post-translational modification, SNO regulates the localization and activity of many key enzymes and receptors [19,27,33], leading to modulation of many canonical signaling pathways, synaptic plasticity, axonal elongation, movement of proteins to the cell membrane, and protein assembly [19,27]. We showed that a low dose of SA in drinking water led to the rapid release of NO and the formation of protein S-nitrosylation, which has previously been identified as a new molecular and cellular mechanism for inhibition of neuronal development [20,34]. Notably, the inhibitory effect of SNO by NPA required 4–6 days, possibly due to the slow turnover rates of S-nitrosylated proteins. The findings above demonstrate that Ca^2+^ and nNOS regulate SNO expression. We suggested that low doses of SA may lead to an imbalance of acetyl-CoA and synaptic homeostasis via SNO. Several reports have shown that arsenic reduces the cholinergic system activity by increasing mRNA expression of acetylcholinesterase [7], reducing the expression of choline acetyltransferase protein and muscarinic receptor activity [35]. Mitochondrial acetyl-CoA-acetyltransferase was S-nitrosylated in mice watered with both 0.1 and 1 ppm SA on the catalytic subunit cysteine 410 [36], which inhibits its activity similar to other enzymes with cysteine at the catalytic site [37,38]. Thereby, we suggest that it may lead to inhibition of acetyl-CoA production (Figure 6).

Importantly, cortical acetylcholine signaling shapes neuronal circuit development and underlies specific aspects of cognitive functions and behaviors, including attention, learning, memory and motivation [39,40]. Vesicle-fusing ATPase (NSF) protein, which is part of the synaptic vesicle release machinery, was SNOed in SA-treated mice. SNO-NSF inhibits the disassembly of the SNARE complex [41] and therefore inhibits exocytosis (Figure 6). Rab GTPase, which regulates vesicle tethering to target membranes, was also SNOed. SNO-Rab facilitates Rab’s GTPase activity [27] leading to an increase of vesicle docking and fusion and, therefore, to exocytosis (Figure 6). Altogether, this may lead to imbalance and alterations in synaptic vesicle cycle and synaptic homeostasis (Figure 6), which are common biological processes associated with diverse behavioral pathologies [42,43].

Watering of mice at 1 ppm of SA promoted protein nitration and apoptosis of neurons in vitro. However, apoptosis was prevented by the inhibition of nNOS. Ntyr, as well as peroxynitrite, are products of the NO-related biochemical reactions. The formation of these molecules results in nitration of proteins under conditions of NO overproduction [44]. Furthermore, an increased level of nitrosative stress and upregulated Ntyr formation could potentially be cytotoxic, leading to neuronal loss in certain neuron types [32]. We further tested GSNO and found a significant upregulation in the treated mice. GSNO forms, at diffusion-controlled rates, radical recombination between NO and GSH thiol radicals (RS), and can then transnitrosate other thiols on peptides and proteins [14,45,46].

In the recovery experiments, we found that the GO analysis of biological processes differs significantly between each of the exclusive sets of the treatment and vs. recovery groups, which supports our conclusions that arsenic-free watering for 10 days reverses the differences in NO biochemistry (see Table S4). Systems biology analysis showed general biological processes for the recovery compared to synaptic and neuronal processes following treatment. In our analysis, we focused on the proteins exclusive for each group in order to analyze only the proteins of treated mice. Proteins overlapping with the control were excluded. It was found that the number of exclusive proteins in treated mice following the recovery period was lower than after the treatment and very similar to control group both before and after the treatment. Next, we compared the Venn Diagrams and found that the overlapped proteins in the treatment groups were lower than those of the recovery groups, which indicates reversibility.

In conclusion, arsenic in drinking water or food may affect the brain adversely and our proteomics and biochemical experiments in mice demonstrate that environmental levels of arsenic induced dose-dependent biochemical changes. This was true both for toxic effects and effects on the neuronal signaling. Mechanistically, arsenic induced a release of calcium that activated formation of NO by nNOS, and promoted nitrosative modifications of proteins. Finally, removal of arsenic exposure reversed S-nitrosylation of the proteome to a “normal” SNO-proteome, suggesting that some neuropathologies that act through overproduction of NO might be ameliorated by reduction of those exposures.

## 4. Materials and Methods

### 4.1. Animals

All animal experiments were performed according to protocols approved by the Massachusetts Institute of Technology Committee on Animal Care and with the National Institutes of Health Guide for the Care and Use of Laboratory Animals (approval code: 0516-035-19; approval date: 1 June 2015). Briefly, *C57BL/6NTac* mice (Taconic laboratories) were used to study the effect of SA (Thermo Fisher Scientific, Waltham, MA, USA, 7784-46-5) on brain neurological function. Mice were divided into three groups, the control group and the arsenic-dosing groups with 0.1 particles per million (ppm) or 1 ppm for 1 month. Each group included twenty animals, with half (10 mice) being used in immunohistochemical analysis and half (10 mice) being used in mass spectrometry analysis. For the recovery experiments, after one month of treatment, all three groups received SA-free sucralose water for 10 days and then were sacrificed. SA solution was prepared as a stock solution at 50 ppm in filter-sterilized water and further diluted to the concentration of 1 ppm in sucralose water (ClearH_2_O). Both sucralose water (control group) and arsenic-containing sucralose water (test group) were provided to mice *ad libitum* for 4 weeks. The animals were kept in a pre-designed “Hazardous Material” area in animal facility and otherwise received standard husbandry care. To maintain the freshness, the drinking water was changed every half a week until necropsy. The gap between the last dose and the necropsy was about 3.5 days. The animals were euthanized using carbon dioxide (CO_2_) inhalation method. Mouse brains were then snap-frozen in liquid nitrogen and kept at −80 °C for further MS analysis or embedded in paraffin blocks for immunohistochemical analysis, as described below.

For anatomy, the mouse skull was incised along a “Y”-shaped cutting line bilaterally on the skull base and the middle line on the rostral surface, and the brain was carefully isolated from the skull.

### 4.2. Primary Cultures of Mouse Embryonic Cortical Neurons

Culture of primary cortical neurons from embryonic mouse was performed according to a previously described protocol [47]. Briefly, 500,000 dissociated embryonic cortical neurons from embryonic day 19 (E19) *Swiss Webster* pregnant mice were plated onto poly-D-lysine coated 12-well plates with or without coverslips and grown in neurobasal medium with B27 supplement (Thermo Fisher Scientific, Waltham, MA, USA, 17504044), 1 mM glutamate, and 1% penicillin/streptomycin sulfate (Thermo Fisher Scientific, Waltham, MA, USA, 15140722). The cells grown on coverslips were harvested for immunostaining or SNOTRAP staining. The cells grown on plates were harvested for calcium dye Fluo-4 (Thermo Fisher Scientific, Waltham, MA, USA, F14201) staining.

Timed-pregnant mice (E16 *Swiss Webster* mice) were purchased from Charles River. The timed-pregnant mice were acclimated for 3 days and then euthanized at E19. During the acclimation time, the mice were maintained in the animal facility of the Division of Comparative Medicine (DCM) at MIT with standard husbandry provided by facility professionals (temperature/humidity, light cycle, water, and diet supply). The euthanizing was performed using CO_2_ inhalation and reassured by cervical dislocation, for the reason that the pregnant mice are more tolerant to hypoxia. The torso cavity was incised open. The fetus was extracted from uterus and decapitated. Each fetus head was transferred into a petri dish, the skull was opened with a fine tweezer and the cerebral cortex was carefully dissected out using 2 fine tweezers under a head-mounted surgical microscope. The collected cortical tissue was grossly cut into small pieces and digested using papain by incubation in water bath at 37 °C. This allowed dissociation of the embryonic cortical neurons into single cells. For primary neuron culture, 5 × 10^5^ dissociated embryonic cortical neurons from E19 fetuses from *Swiss Webster* pregnant mice were plated onto poly-D-lysine coated 12-well plates with (for immunocytochemistry and SNOTRAP staining) or without (for other experiments, such as calcium staining by Fluo4, read by plate-reader) coverslips. The plating media contained neurobasal medium, 10% FBS, 1% glutamate, and 1% penicillin/streptomycin. After 1 day, the plating media was changed into regular neurobasal medium with B27 supplement, 1 mM glutamate and 1% penicillin/streptomycin sulfate. The cells were grown for maximally 14 days (DIV14). To maintain sufficient growth of neuron cells, fifty percent of the culture medium was changed every 3.5 days. The arsenic treatment would be carried out in these cultured primary neurons, from after culture day 7 (DIV7) until culture day 14 (DIV14). Later, the cells grown on coverslips were harvested for immunostaining or SNOTRAP staining. The cells grown on plate were harvested for calcium dye Fluo-4/AM staining.

### 4.3. Treatment of Primary Culture Neurons with SA

After plating, the primary culture neurons were grown for 7 days, checked each 3 days to observe maturation with sufficient neuronfiber formation, and next cells were treated with either 0.1 or 1 µM of SA dissolved in ddH_2_O and further cultured for additional 1–6 days. As in the aforementioned protocol, in DIV1, the neurobasal medium was added to replace the plating medium and on DIV4, 50% of culture medium was changed with fresh medium.

From DIV7 on, cells were treated with either 0.1 or 1 µM of SA dissolved in ddH_2_O (SA being prepared as 0.05 mM or 0.5 mM stock solution and 2µl stock solution was used per treatment) and further cultured for additional 1–6 days. The negative control samples were treated with equal amount of ddH_2_O. When the culture time was longer than 3 days, the medium change (50% of medium) was carried out as mentioned in previous sections and SA of the desired concentration was also included in the changing medium. At 1, 2, 4, and 6 days post treatment, cells on coverslips were harvested for further analysis. The number of replicates were at least 4, with >2 rounds of experiment and 2 replicates per experiment being included.

### 4.4. Synthesis of SNOTRAP-Biotin

To stirred 2-(diphenylphosphino)-benzenethiol (2,3) (100 mg, 0.34 mmol) in dry DMF (5 mL), biotin-PEG3-propionic acid (100 mg, 0.22 mmol, ChemPep, Inc, Wellington, FL, USA, 52881-76-8), N,N’-dicyclohexylcarbodiimide (DCC) (70 mg, 0.34 mmol) and dimethylaminopyridine (DMAP) (4 mg, 0.03 mmol) were added successively. The resulting mixture was stirred for 7 h at room temperature, and the resulting clear solution was then concentrated under reduced pressure and purified by flash chromatography (hexane/EtOAc/MeOH gradient) to give the desired product (yield 30%). The SNOTRAP probe was repurified on an 1100 HPLC system with a photodiode array UV detector at 254 nm (Agilent Technologies, Wilmington, DE). For detailed protocol see [21].

### 4.5. Chemical Staining of S-Nitrosylation Using SNOTRAP Probe

S-nitrosylation was detected via chemical staining method using the SNOTRAP probe. SNOTRAP was prepared as described previously [48] and conjugated with either fluorophore (Cy5) (Thermo Fisher Scientific, Waltham, MA, USA) or a biotin molecule. Upon harvest, primary cultured neurons were grown on a coverslip, washed twice with warm 1× phosphate-buffered saline (PBS) (Thermo Fisher Scientific, Waltham, MA, USA, 10010023) and then fixed with 100% methanol at −20 °C for 15 min. Cells were then incubated with 300 nM N-ethylmaleimide (NEM) (Thermo Fisher Scientific, Waltham, MA, USA, 23030) in PBS-Triton ×100 (0.3% *v*/*v*) at 37 °C for 30 min to block the free sulfhydryl groups on proteins. The NEM solution was freshly prepared. First, it was dissolved in a small amount (10% of total volume) of methanol, and then further dissolved in PBS-Triton ×100 (0.3% *v*/*v*). Cells were then washed 3 × 5 min using 1× PBS and incubated with SNOTRAP probe (250μM) in acetonitrile-PBS-Triton (50%: 50% *v*/*v*) at room temperature (R.T.) for 1 h to allow binding of SNOTRAP with –SNO groups on proteins. Cells were then washed 5 × 10 min, counterstained with DAPI (Thermo Fisher Scientific, Waltham, MA, USA, D1306), and mounted with SlowFade^®^ Gold antifade medium (Invitrogen). Alternatively, when biotin-conjugated SNOTRAP was used, cells were washed 5 × 10 min after SNOTRAP treatment, then further incubated with streptavidin-DyLight 488 (Thermo Fisher Scientific, Waltham, MA, USA, 21832) diluted 1/500 with PBS-Triton × 100 (0.3% *v*/*v*), incubated at R.T. for 1 h and afterwards washed 5 × 5 min. To obtain negative control samples, after fixation, cells were pre-treated with freshly prepared ascorbic acid 20 mM in 1× PBS-Triton × 100 at R.T. for 1 h. This allowed de-S-nitrosylation [21]. Alternatively, de-S-nitrosylation was achieved by exposing cells to UV light [21] at R.T. for 1 h. An additional negative control sample was prepared by incubating cells with solutions containing only the Cy5 dye without the SNOTRAP probe.

### 4.6. Pharmacological Experiments

For N-methyl-D-aspartate (NMDA) (Sigma, St. Louis, MO, USA, M3262) excitotoxicity experiments, primary culture neurons were treated with 10–40 µM of NMDA (kept in 100 mM/mL stock solution in water and further dissolved in neurobasal medium for desired concentration) for 60 min to initiate toxicity, changed with normal culture medium for recovery and harvested 24 h after the treatment to observe and compare the levels of SNO signal. To test the inhibitory effect of NPA (Sigma, St. Louis, MO, USA, SML2341), primary culture neurons were treated with 2 µM nNOS inhibitor NPA (kept in 0.5mM stock solution in water; 4µ was used) for 2, 4, and 6 days. The levels of SNO signal were measured using SNOTRAP staining. To test whether the calcium channel blocker 2-APB (Cayman Chemistry, Ann Arbor, MI, USA, 14538) would bring any inhibition of SNO signaling, 100 µM 2-APB was used to co-treat the cells along with 1 µM SA for 6 days. The 2-APB was dissolved in DMSO (100%, Sigma, St. Louis, MO, USA, D4540) to make 2 mM stock solution. For the treatment, 1µl of the 2-APB stock solution was used per well of a 24-well plate with 1 mL culture medium. For control group, only 1 µl DMSO was applied. The levels of SNO signal were compared between groups with and without 2-APB co-administration. The number of replicates in the above-mentioned experiments was at least 4, with >2 rounds of experiment. Two replicates were included in each experiment. Researchers have shown that 2-APB is stable for at least two days [49]. Therefore, the stability of 2-APB should be durable and lasting long enough to exert certain interactions with SA and nNOS.

### 4.7. Immunocytochemistry of Primary Culture

For immuno-staining of nitrotyrosine, primary cultured neurons were fixed with 100% methanol at −20 °C, briefly rinsed with 1× PBS, blocked with 3% BSA in PBS-Triton-X100 (0.1% *v*/*v*) for 1 h, incubated with primary nitrotyrosine antibody (Millipore, 05-233) (1/200 dilution) for 1 h at R.T., and washed 3 × 5 min with 1×PBS-Tween20 (0.05% *v*/*v*). This was followed by the incubation with secondary antibody (Invitrogen, Carlsbad, CA, USA, 1/500 dilution) for 1 h at R.T. Then, the neurons were washed 5 × 5 min with 1× PBS-Tween20 (0.05% *v*/*v*), counterstained with DAPI and mounted with SlowFade^®^ Gold antifade medium (Invitrogen, Carlsbad, CA, USA, S36936).

### 4.8. Immunohistochemistry of Brain Sections

Immunohistochemical staining of mouse brain sections was performed according to standard protocol. After necropsy, the brain was carefully dissected and isolated from the skull bone, fixed with 10% formalin solution at 4 °C overnight and then sliced carefully with freehand. The position to perform the coronal sectioning, and after sectioning, each section was recognized for the brain functional areas according to the reference/annotation resource from the http://mouse.brain-map.org/static/atlas/ (“Coronal atlas”). Appropriately, the same or similar section levels were used for further histological examination. We were focused on the planes of (1) the prefrontal cortex-stratium; (2) the motor cortex-somatosensory cortex, and (3) the hippocampus. The coronal brain slices were embedded in paraffin blocks and cut into 4 μM sections. Before carrying out immunohistochemical staining, the formalin-fixed paraffin-embedded (FFPE) tissue sections were in parallel H&E-stained to examine that the appropriate brain functional areas were all present for analysis. The FFPE tissue sections were deparaffinized in 100% Xylene (Sigma St. Louis, MO, USA, 95660) and underwent a series of washes in 100%, 90% and 70% ethanol and ddH_2_O. Then, the tissue sections were incubated with citrate-based antigen retrieval buffer (Dako, pH = 6.0) at 95 °C for 20 min, washed in 1× PBS and blocked with 3% BSA (Sigma, St. Louis, MO, USA, A2058) in PBS-Triton X100 overnight. After that, the tissue sections were incubated with nitrotyrosine primary antibody (Millipore, 05-233, diluted 1:200) for 1 h at R.T., washed in PBS-Tween 20 (0.05% *v*/*v*) and incubated with secondary antibody (goat-anti-rabbit) (Invitrogen, Carlsbad, CA, USA, A32731) at R.T. for 1 h. The sections were washed in PBS-Tween 20 and counterstained with DAPI. We tried to stain the SNO in the thicker (20 or 10 μm) brain sections. Firstly, it appeared that the NEM and the SNOTRAP were not able to penetrate deep enough into the tissues, only leaving an extreme strong signal on the tissue surface. Secondly, it was found out that the background autofluorescence was quite high. We did not proceed with the thinner 4 μm FFPE sections because paraffin melts at 55 °C and this could destroy any redox signal.

### 4.9. Fluo-4/AM Staining of Calcium

To determine changes of Ca^2+^ by SA, the Ca^2+^-sensing Fluo-4/AM dye was used. Briefly, Fluo-4/AM (from the 2 mM stock solution dissolved in 100% DMSO) was applied to primary culture neuron cells at a final concentration of 2 μM. The cells were incubated with Fluo-4/AM at 37 °C for 1 h. The excess dye was removed by washing twice in PBS. Afterwards, SA (0.1 ppm or 1 ppm) was added to the medium and plates were read using a Cellomics plate reader. Fluorescent images were collected with excitation/emission wavelength of 494/506 nm and captured using standard FITC filters, as being instructed by the manufacturer, at R.T. every 30 s for up to 2 h.

### 4.10. TUNEL Assay

TUNEL assay (CN: 17141/Millipore) was carried out according to the manufacturer’s instructions. For FFPE sections, samples were deparaffinized and rehydrated with ethanol wash. Next, sections were incubated with 20 µg/mL proteinase K (Thermo Fisher Scientific, Waltham, MA, USA, AM2548) for 20 min at 37 °C to remove formalin-generated crosslinks. For cultured neurons on coverslips, cells were fixed with methanol. Briefly, the samples were treated with TdT enzyme (Thermo Fisher Scientific, Waltham, MA, USA, EP0161), washed, and incubated with anti-digoxigenin conjugate to label apoptotic cells.

### 4.11. Morphometric Analysis

Image Pro-Plus 7.0 ^®^ system (Media Cybernetics, Silver Spring, MA, USA) or ImageJ 2.0 [50] was used for image analysis. They were recorded by QIClick^®^ digital CCD camera (Q Imaging, Surrey, BC, Canada) mounted on top of a conventional light microscope (Zeiss Axioskop 2plus). For immunohistochemical analysis, each brain area of interest, such as prefrontal cortex, motor cortex, and somatosensory cortex, was represented by a series of 20× continuous pictures with equal exposure from cortical surface to corpus callosum incorporating the entire depth of cortical layers. Typically, for the prefrontal cortex, 2–3 pictures and for the motor and somatosensory cortex, 4–5 pictures were required. Each set of pictures was stitched together and analyzed for its size, cell number, and fluorescent intensity (greyscale) of nitrotyrosine staining. For the quantification of greyscale fluorescent intensity, each picture was subjected to background subtraction using “rolling-ball background subtraction”, and the total grayscale value was calculated. For the quantification of the cell number, the numbers of positive DAPI counterstained cells were counted. The total grayscale intensity value was further equalized using the cell number justification. For each animal, 6–8 sets of pictures were used and the immunostaining value was represented as the average. For immunocytochemical or SNOTRAP chemical staining analysis, each sample (either untreated or treated with SA) was represented by 8–10 independent 20× pictures and analyzed for its cell number and fluorescent intensity. Since the data distributed normally, statistical analyses were performed using a Student’s t test with *p*-value cutoff of *p* < 0.05. Data were expressed as average fluorescent intensity per cell.

### 4.12. Materials and Reagents for Mass Spectrometry

Biotin-PEG3-propionic acid was purchased from ChemPep Inc. and protease cocktail inhibitors from Sigma (St. Louis, MO, USA). Sequencing-grade modified trypsin was bought from Promega. The high-performance liquid chromatography (HPLC) and the liquid chromatography-mass spectrometry (LC-MS) solvents were of HPLC grade. Acetonitrile (ACN) and distilled water for MS were acquired from Sigma (St. Louis, MO, USA). Vivapsin 10k molecular weight cut off (MWCO) filters were purchased from Sartorius corporation, Cat. No. VS15T01. Synthesis of SNOTRAP-biotin and NMR analysis are described in details in ref [21]. All sample preparation was conducted in the dark at R.T.

### 4.13. GSNO Analysis

The quantification of GSNO internal standard synthesis in the cortex, sample preparation for GSNO quantification, and ESI +/− QqQ-MS analyses were carried out as described by us previously [22]. Briefly, an online coupling of solid-phase derivatization (SPD) with liquid chromatography–mass spectrometry (LC-MS) was developed and applied in the analysis of GSNO. GSNO was quantified using this automated online method with good linearity (*R*^2^ = 0.9994); the limit of detection was 0.015 nM. The online SPD-LC1MS method has been used to determine GSNO levels in mice cortex

### 4.14. Inductively Coupled Plasma Mass Spectrometry (ICP-MS) Analysis of SA

Digestion of tissues for inductively coupled plasma mass spectrometry. Frozen samples were weighted, allowed to thaw on ice and added directly to the bottom of a teflon digestion container. 2.0 mL of nitric acid (Sigma, St. Louis, MO, USA, 695041) was added to the tissue. Using the ethos up microwave digestion system (Milestone Srl., Sorisole, Bergamo, Italy). Samples were heated to 93 °C over 15 min, then to 104 °C over 10 min, and allowed to cool. All samples were visually inspected to be clear with no particulate and transferred to 50 mL polypropylene falcon tubes. The contents of the teflon digestion tubes were rinsed twice with 1.0 mL of 1% nitric acid and stored until ICP-MS analysis. The multiple reaction monitoring (MRM) mode of a QqQ MS was used to establish a sensitive and selective quantification method.

### 4.15. Homogenization and Preparation of Brain Tissue for Mass Spectrometry 

The whole cortex was dissected from 8-week-old control and arsenic-treated mice, removed and transferred in liquid nitrogen to store in −80 °C until use. Two biological replicates and 3 technical replicates for each biological replicate were used. Each biological replicate for the cortex was a pooling of 3 cortex tissues from 3 mice. Replicates from each group were pooled and used for analysis.

The tissue was homogenized with lysis buffer, containing: 250 mM HEPES-NaOH, pH 7.7, 1 mM EDTA, 0.1 mM neocuproine (Sigma, St. Louis, MO, USA, N1501), 1% NP-40 (Merck, Kenilworth, NJ, USA, 127087-87-0), 20 mM IAM (Merck, Kenilworth, NJ, USA, 144-48-9), and 1% protease inhibitor cocktail (Sigma, St. Louis, MO, USA, P8340). The homogenates were centrifuged at 12,000 *g* for 10 min at 4 °C and then the supernatant was collected. We determined the protein concentration by a Bradford assay (Bio-Rad, Cat. No. 500-0006). The negative controls were generated by treatment with 10 mM TCEP (Thermo Fisher Scientific, Waltham, MA, USA, 20491) for 30 min at 37 °C after sample mixing. Samples were then alkylated with 30 mM IAM in the presence of 2.5% SDS in the dark at 37 °C. Then, samples were twice washed with 3 times volumes of 8 M Urea (in 50 mM HEPES, pH 7.7) and once with 50 mM HEPES (pH 7.7) by centrifugation at 5,000 *g* for 30 min at 4 °C with 10 K MWCO spin filters (Sartorius corporation, VS15T01). We labeled the proteins using SNOTRAP stock solutions (in 40% ACN) that were added to all samples to reach a final concentration of 1.25 mM and to selectively convert SNO to stable disulfide-iminophosphorane. The samples were incubated with SNOTRAP solution at R.T. for 1 h. Then, excess reagents were removed by three washes with 50 mM HEPES, pH 7.7 buffer with 10 K filters. After filtration, 200–300 μL pre-rinsed Streptavidin agarose beads (Pierce, Appleton, WI, USA, 20349) were added to each sample and incubated for 1 h at R.T. with gentle shaking. The beads were washed with washing buffer (50 mM HEPES, 150 mM NaCl, 0.1% SDS, pH 7.7) three times and then with washing buffer (50 mM HEPES, pH 7.7) three times. Proteins were eluted with 10 mM TCEP (in 50 mM HEPES, pH 7.7) and alkylated with 10 mM IAM. Then, samples were trypsinized (Promega, San Luis Obispo, CA, USA, V5111) at 37 °C for 4 h. Finally, the samples were desalted with C18 StageTips as described earlier [51].

### 4.16. Mass Spectrometry Analysis

We analyzed the trypsinized peptides using a QExactive MS coupled to an Easy-nLC 1000 (Thermo Fisher Scientific, Waltham, MA) interfaced via a nanoSpray Flex ion source. We used the C18 column (75 μm ID × 250 mm, 2 mm, Thermo Fisher Scientific, Waltham, MA, USA).

We used water (0.1% Formic Acid (FA)) and ACN (0.1% FA) as A and B mobile phases, respectively. We dissolved samples in 0.1% FA, and injected to a C18 pre-column (75 μm ID × 20 mm, 3 µm, Thermo Fisher Scientific, Waltham, MA, USA) and separated by a C18 column (75 μm ID × 250 mm, 2 µm, Thermo Fisher Scientific, Waltham, MA, USA) with a linear gradient (1% B for 10  min, 1–60% B for 110  min, and 60–100% B for 10  min) followed by a 10 min post-run at 1% B at a flow rate of 300  nL/min. MS spectra were acquired in data-dependent mode using the following settings: spray voltage, 2.2 kV; capillary temperature, 250 °C; S-lens RF level, 60%; no sheath and auxiliary gas flow; resolution 70,000; scan range 350–1800 Th. Up to the top 10 most abundant ions with multiple-charged ions from the full MS scan were selected with an isolation window of 2 and fragmented by higher-energy collision dissociation with normalized collision energies of 28 at a resolution of 35,000. The maximum ion injection times for the full MS scan and the MS/MS scan were both 100 ms. The ion target values for the full scan and the MS/MS scans were set to 3 × 10^6^ and 1 × 10^5^, respectively. Xcalibur software was used for data acquisition. Two technical runs were conducted for each sample.

Peptides digests were also analyzed on an Agilent 6550 Nano-HPLC-Chip/MS system, consisting of a micro-autosampler, a capillary and nanoflow pump, and the Chip-Cube that interfaces LC modules and the MS instrument. We used water (0.1% FA) and ACN (0.1% FA) as A and B mobile phases, respectively. The separation of the peptides were carried out on a Polaris-HR-Chip-3C18 HPLC-Chip (Agilent Technologies, Santa Clara, CA, USA, G4240-62030). It consists of a 360 nL enrichment column and a 75 μm× 150 mm analytical column; both were packed with Polaris C18-A, 180A, 3 μm stationary phase. The peptides were loaded onto the enrichment column from the autosampler at a constant flow of 2 μL/min. A 55 min gradient initiated at 3% B at 300 nL/min and increased to 30% B from 2 to 35 min, to 60% B at 40, to 90% B at 45 min and then was held for 5 min and followed by a 5 min postrun at 3% B. The positive-ion MS spectra was acquired in the 1700 Da extended dynamic range mode (2 GHz) using the following setting: ESI capillary voltage, 1960 V; fragmentor, 360 V; Octopole RF peak, 750 V; drying gas, 13 L/min; drying temperature, 225 °C. The data were acquired at a rate of 6 MS spectra per second and 3 MS/MS spectra per second in the mass range of m/z 300–1700 for MS and 50–1700 for MS/MS. The parameters were set on maximum number of precursors per cycle was 20, with a threshold of 5000 ions in a precursor abundance-based scan speed in peptide isotope model, with +2, +3 and higher charge-state preference, and with active exclusion after one spectrum and released after 0.15 min. Applying fragmentation energy was done at a slope of 3.1 V/100 Da with a 1.0 offset for doubly charged precursors, 3.6 V/100 Da with a −4.8 offset for triply and multiply charged precursors. Maintaining the mass accuracy by using internal reference ion *m/z* 1221.9906. MassHunter Workstation software (Agilent, Agilent Technologies, Santa Clara, CA, USA) was used for data acquisition.

### 4.17. MS Data Processing

We used the Agilent Spectrum Mill MS proteomics Workbench B.05 for the following: peak list generation, database searching, and FDR estimation.

We set the following parameters for extraction: cysteine carbamidomethylation for fixed modification, precursor MH + 300–8000 Da, scan time range 0–200 min, sequence tag length > 1, merge scans with the same precursor m/z +/− 30 s and same +/− 0.05 m/z, default for precursor charge, true for find 12C precursor, MS noise threshold 100 counts. We searched the MS/MS spectra against the mouse SwissProt protein database (downloaded on 11/01/2016, 16,813 items) with +/− 20 ppm precursor ion tolerance and +/− 50 ppm fragment ion tolerance. This included variable modifications of methionine oxidation, protein N-terminal acetylation, deamidation of asparagine, and fixed modification of cysteine carbamidomethylation. The FDR was set to 1.2% for peptide identification and protein polishing, The MS data (raw and search data of each replicate) have been deposited to the ProteomeXchange Consortium via the PRIDE [52] partner repository with the dataset identifier PXD012691.

### 4.18. Bioinformatics and Statistics

GO processes and networks were generated after submitting the lists of SNO-proteins to “MetaCore from Thomson Reuters”, MetaCore™ version 6.34 build 69,200. We used Benjamini-Hochberg correction [53] on the *p*-value to generate FDR, and processes/terms with FDR values below 0.05 were included. We used STRING (version 10.0) to analyze the protein-protein interaction of SNO-proteins (http://string-db.org) [54].

## Data Availability

Raw and search data have been deposited to the ProteomeXchange Consortium via the PRIDE partner repository with the dataset identifier PXD012691.

## Supplementary Materials

Supplementary materials can be found at https://www.mdpi.com/1422-0067/21/11/3948/s1.

## Abbreviations

ACN	Acetonitrile
DOAJ	Directory of open access journals
FA	Formic Acid
FDR	False discovery rate
FFPE	Formalin-fixed paraffin-embedded
GSNO	S-nitrosoglutathione
HPLC	High-performance liquid chromatography
ICP-MS	Inductively Coupled Plasma Mass Spectrometry
LC-MS	Liquid chromatography–mass spectrometry
LD	Linear dichroism
MDPI	Multidisciplinary Digital Publishing Institute
MS	Mass spectrometry
MWCO	Molecular weight cut off
NO	Nitric oxide
NPA	N(ω)-propyl-l-arginine
Ntyr	3-nitrotyrosine
SA	Sodium arsenite
SNO	S-nitrosylation
SPD	Solid-phase derivatization
TdT	Terminal deoxynucleotidyl transferase
TLA	Three letter acronym
TUNEL	Terminal deoxynucleotidyl transferase dUTP nick-end labeling
dUTP	2´-Deoxyuridine, 5´-Triphosphate
2-APB	2-Aminoethoxydiphenyl borate
