# Supplementary material for: Low Doses of Arsenic in a Mouse Model of Human Exposure and in Neuronal Culture Lead to S-Nitrosylation of Synaptic Proteins and Apoptosis via Nitric Oxide
Source: Int J Mol Sci. 2020 May 31;21(11):3948. doi: 10.3390/ijms21113948 (PMC7312481; doi:10.3390/ijms21113948)
Supplement: Supplementary file 1 [file ijms-21-03948-s001.zip › Supp Info/Supplementary Materials_IJMS.pdf]

Supplementary Materials for

Low doses of arsenic in a mouse model of human exposure and in neuronal culture lead to S-nitrosylation of synaptic proteins and apoptosis via nitric oxide

Haitham Amal [1*†], Guanyu Gong[2†], Hongmei Yang[2], Brian A. Joughin[2,3], Xin Wang[2], Charles G. Knutson[2], Maryam Kartawy[1], Igor Khaliulin[1], John S. Wishnok[2], and Steven R. Tannenbaum[2,4*]

[1] Institute for Drug Research, School of Pharmacy, Faculty of Medicine, The Hebrew University of Jerusalem, Jerusalem 91120, Israel
[2] Department of Biological Engineering, Massachusetts Institute of Technology, Cambridge, MA 02139, USA
[3] Koch Institute for Integrative Cancer Research, Massachusetts Institute of Technology, Cambridge, MA 02139, USA
[4] Department of Chemistry, Massachusetts Institute of Technology, Cambridge, MA 02139, USA

[†] HA and GG have contributed equally to this study.

[*] Corresponding Authors: srt@mit.edu, haitham.amal@mail.huji.ac.il
Address: Massachusetts Institute of Technology, 77 Massachusetts Avenue, Room 56-731A Cambridge, MA 02139. Phone: +1-617- 253-3729. Fax: +1-617-252-1787

Figures S1 – S4

Captions for Tables S1 – S4

Caption for Movie S1

Figs. S1 to S4

Fig. S1:

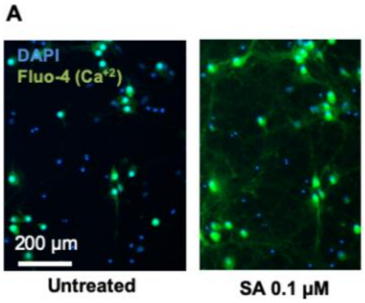

Changes in calcium were studied using Fluo4 (Ca^{+2}) calcium-sensing dye to track calcium after treatment of SA 0.1 µM for 5 minutes using primary cultures of mouse embryonic cortical neurons. Both figures represent the same cells.

Fig. S2:

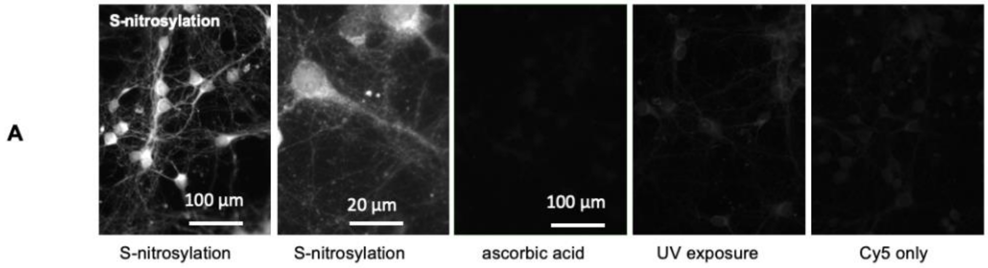

The establishment of SNOTRAP for chemical staining of S-nitrosylation signals in primary neuron culture. A) Primary cultured neuron cells were treated with SNOTRAP-Cy5 (left 2 columns). To show that SNOTRAP binds specifically to the S-nitrosylated proteins, negative control samples were prepared with SNO-reducing reagent (ascorbic acid or UV light) and SNOTRAP-Cy5 (middle 2 columns, as negative control) or Cy5 only (as negative control).

Fig. S3:

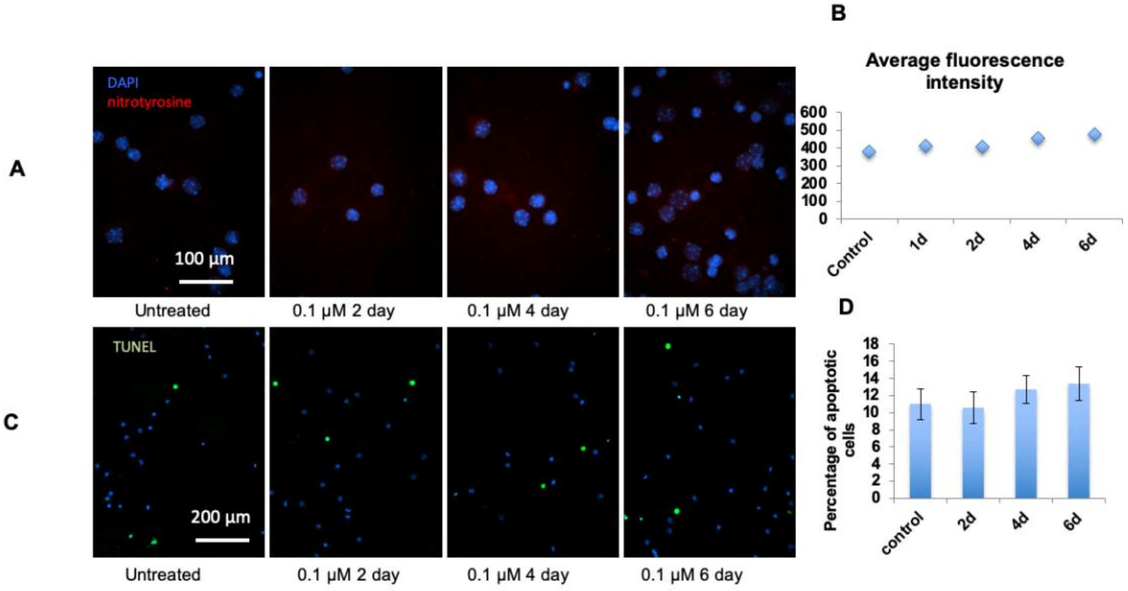

The changes in tyrosine nitration (Ntyr) and apoptosis in cultured neurons was measured following treatment with 0.1 µM SA for 1–6 days. A) The changes of Ntyr were measured using 3-nitrotyrosine immune staining. Quantitative analysis was conducted and is shown on the right; B) no significant difference was found. C) Cell apoptosis was measured using TUNEL assay. Quantitative analysis was conducted and is shown on the right; D) no significant difference was found. All quantification figures were presented as mean and ± SEM. One Way Anova was conducted.

Fig. S4:

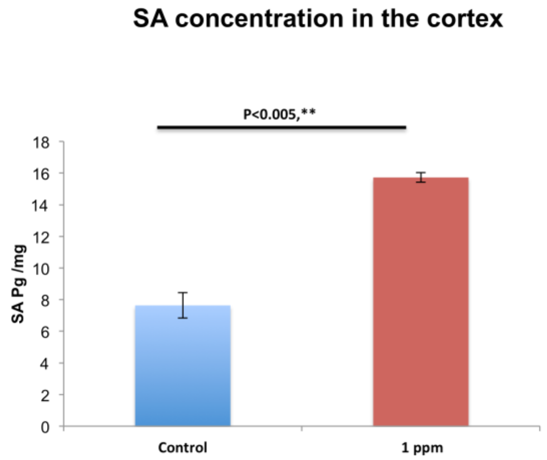

SA concentration in the cortex in control and 1 ppm SA groups. The multiple reaction monitoring (MRM) mode of a triple quadrupole mass spectrometer (QqQ MS) was used to establish a sensitive and selective quantification method. Presented as mean and ± SEM. One tailed t-test was conducted. P<0.005**, control mice (n=5), 1 ppm SA mice (n=5).

Captions for Tables S1 to S4

Table S1: Lists of SNO proteins in the different groups following treatment and after recovery.

Table S2: Gene Ontology analysis of the different groups following treatment and after recovery. Top 30 significant terms.

Table S3: List of SNO proteins and GO analysis of the 35 proteins that are common between SA 0.1 ppm and 1 ppm groups.

Table S4: GO analysis of the exclusively proteins following recovery and treatment of 0.1 ppm and 1 ppm.

Caption for Movie S1

Movie S1: using the calcium-sensing dye Fluo-4/AM, it was found that the concentration of calcium rapidly increased after addition of 0.1 µM SA for 5 seconds.
